# Publisher Correction: Parameters for one health genomic surveillance of *Escherichia coli* from Australia

**DOI:** 10.1038/s41467-025-58600-0

**Published:** 2025-04-07

**Authors:** Anne E. Watt, Max L. Cummins, Celeste M. Donato, Wytamma Wirth, Ashleigh F. Porter, Patiyan Andersson, Erica Donner, Australian Pathogen Genomics One Health Working Group, Vitali Sintchenko, Alicia Arnott, Alireza Zahedi, Rowena Bull, Jessica R. Webb, Danielle Ingle, Kristy Horan, Tuyet Hoang, Angeline Ferdinand, Tehzeeb Zulfiqar, Craig Thompson, Lex E. X. Leong, Bethany Hoye, Glenn F. Browning, Michelle Wille, Rose Wright, Angela Donald, Zoe Bartlett, Avram Levy, Christina Bareja, Tatiana Gonzales, Cara Minney-Smith, Erin Flynn, Aruna Phabmixay, Thy Huynh, Amy V. Jennison, Torsten Seemann, Steven P. Djordjevic, Benjamin P. Howden

**Affiliations:** 1 Microbiological Diagnostic Unit Public Health Laboratory, Department of Microbiology and Immunology at The Peter Doherty Institute for Infection and Immunity, The University of Melbourne, Melbourne, Victoria, Australia; 2 Department of Microbiology and Immunology at The Peter Doherty Institute for Infection and Immunity, The University of Melbourne, Melbourne, Victoria, Australia; 3 Australian Institute for Microbiology and Infection, University of Technology Sydney, Ultimo, New South Wales, Australia; 4 The Australian Centre for Genomic Epidemiological Microbiology, University of Technology Sydney, Ultimo, New South Wales, Australia; 5 Centre for Pathogen Genomics, The University of Melbourne, Melbourne, Victoria, Australia; 6 Future Industries Institute, University of South Australia, Mawson Lakes, South Australia Australia; 7 Cooperative Research Centre for Solving Antimicrobial Resistance in Agribusiness, Food, and Environments (CRC SAAFE), Mawson Lakes, South Australia Australia; 8 Public Health Microbiology, Public and Environmental Health, Pathology Queensland, Queensland Department of Health, Brisbane, Queensland Australia; 9 Department of Infectious Diseases, Austin Health, Heidelberg, Victoria, Australia; 10 Centre for Infectious Diseases and Microbiology Laboratory Services, Institute of Clinical Pathology and Medical Research, NSW Health Pathology, Westmead Hospital, Westmead, New South Wales, Australia; 11 Victorian Infectious Diseases Reference Laboratory, Royal Melbourne Hospital, Doherty Institute for Infection and Immunity, Melbourne, Victoria, Australia; 12 School of Biomedical Sciences, Faculty of Medicine, University of New South Wales, Sydney, New South Wales, Australia; 13 National Centre for Epidemiology and Population Health, The Australian National University, Canberra, Australian Capital Territory Australia; 14 SA Pathology, Adelaide, South Australia Australia; 15 Environmental Futures, School of Earth, Atmospheric and Life Sciences, University of Wollongong, Wollongong, New South Wales, Australia; 16 Asia-Pacific Centre for Animal Health, Melbourne Veterinary School, Faculty of Science, University of Melbourne, Parkville, Victoria, Australia; 17 Office of Health Protection, Australian Government Department of Health and Aged Care, Canberra, Australian Capital Territory Australia; 18 Food Standards Australia and New Zealand, Canberra, Australian Capital Territory Australia; 19 Department of Microbiology, PathWest Laboratory Medicine WA, Queen Elizabeth II Medical Centre, Perth, Western Australia Australia

**Keywords:** Bacterial genetics, Bacterial infection, Microbial ecology, Epidemiology

Correction to: *Nature Communications* 10.1038/s41467-024-55103-2, published online 02 January 2025

In the version of the article initially published, an earlier, incorrect version of Supplementary Data 1 was included. The correct Supplementary Data 1 is now available online.

## Supplementary information

Updated Supplementary Dataset 1

