# Supplementary material for: Parameters for one health genomic surveillance of Escherichia coli from Australia
Source: Nat Commun. 2025 Jan 2;16:17. doi: 10.1038/s41467-024-55103-2 (PMC11696363; doi:10.1038/s41467-024-55103-2)
Supplement: Supplementary file 1 — Supplementary Information File [file 41467_2024_55103_MOESM1_ESM.pdf]

Supplementary Information

Parameters for One Health genomic surveillance of *Escherichia coli* from Australia

Anne E. Watt[^,1,2], Max L. Cummins[^,3,4], Celeste M. Donato[^2,5], Wytamma Wirth[2], Ashleigh F. Porter[2], Patiyan Andersson[1,2], Erica Donner[6,7], Australian Pathogen Genomics One Health Working Group, Amy V. Jennison[8], Torsten Seemann[1,2,5], Steven P. Djordjevic[+*,3,4] and Benjamin P. Howden[+*,1,2,5,9]

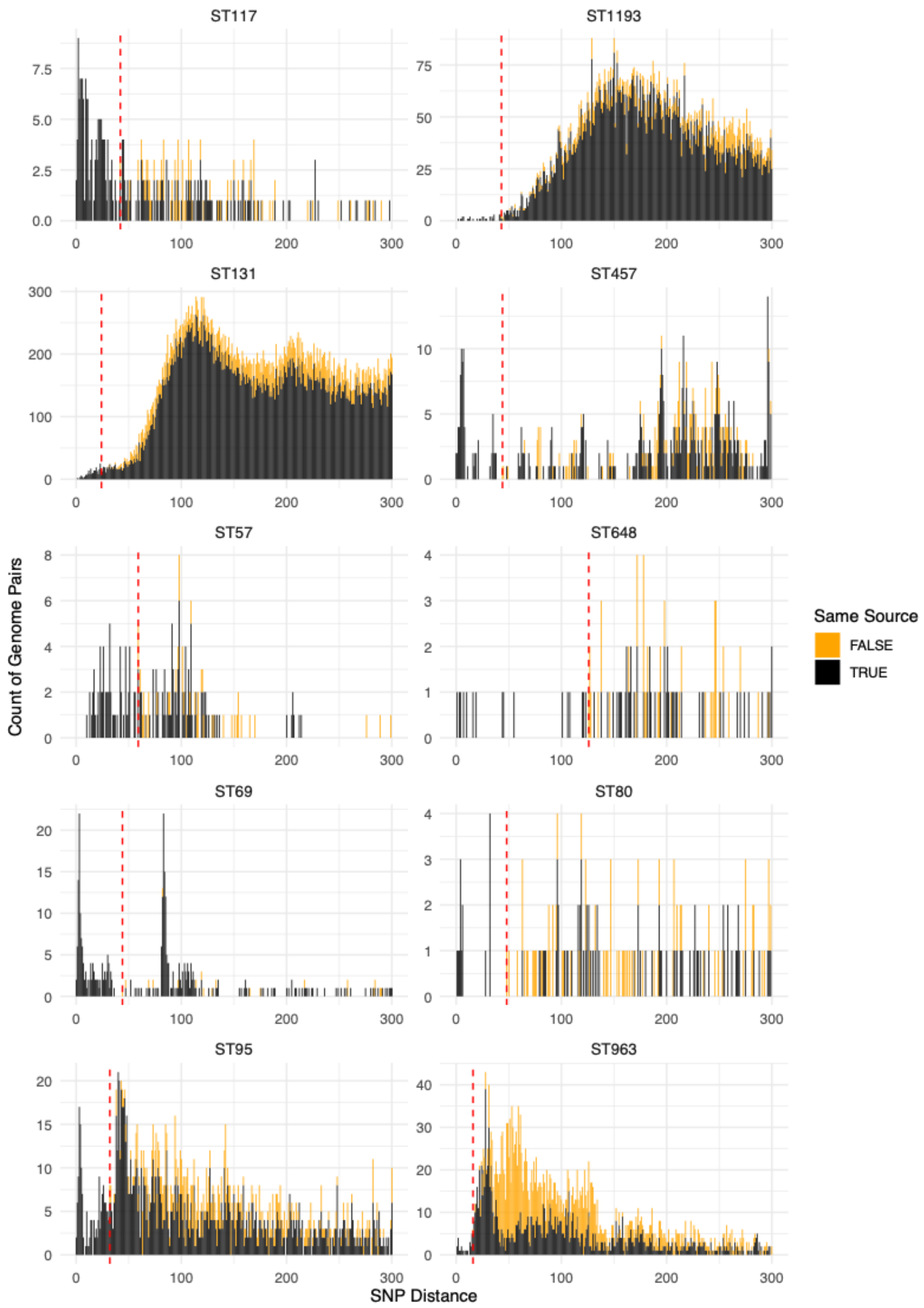

Supplementary Fig. 1. Stacked histograms of pairwise SNPdistances by STgrouped by intrasource and intersource pairs. The red dashed line represents the minimal SNP distance for intersource pairs within a given ST.

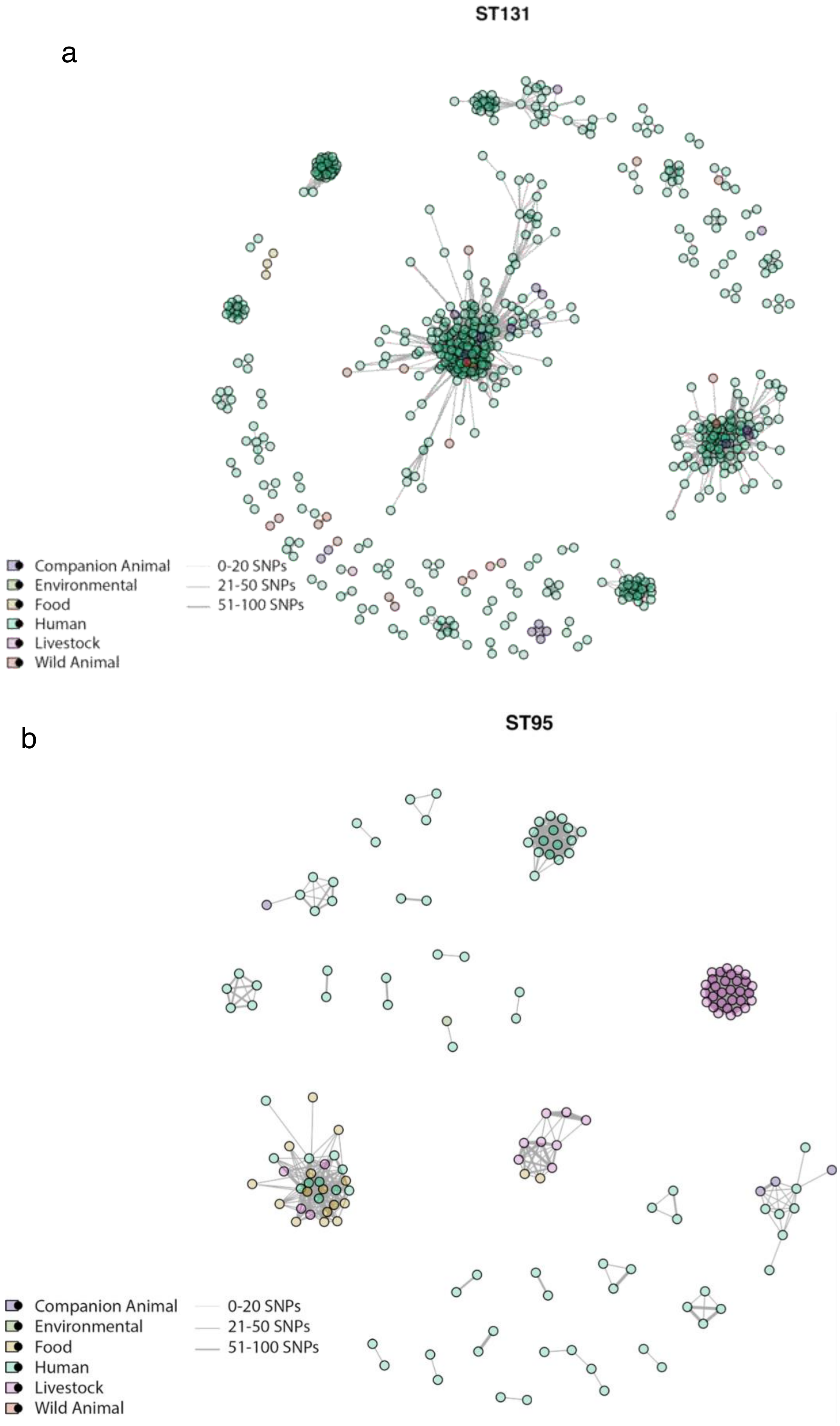
ST131
a
Companion Animal
Environmental
Food
Human
Livestock
Wild Animal
0-20 SNPs
21-50 SNPs
51-100 SNPs
ST95
b
Companion Animal
Environmental
Food
Human
Livestock
Wild Animal
0-20 SNPs
21-50 SNPs
51-100 SNPs

c

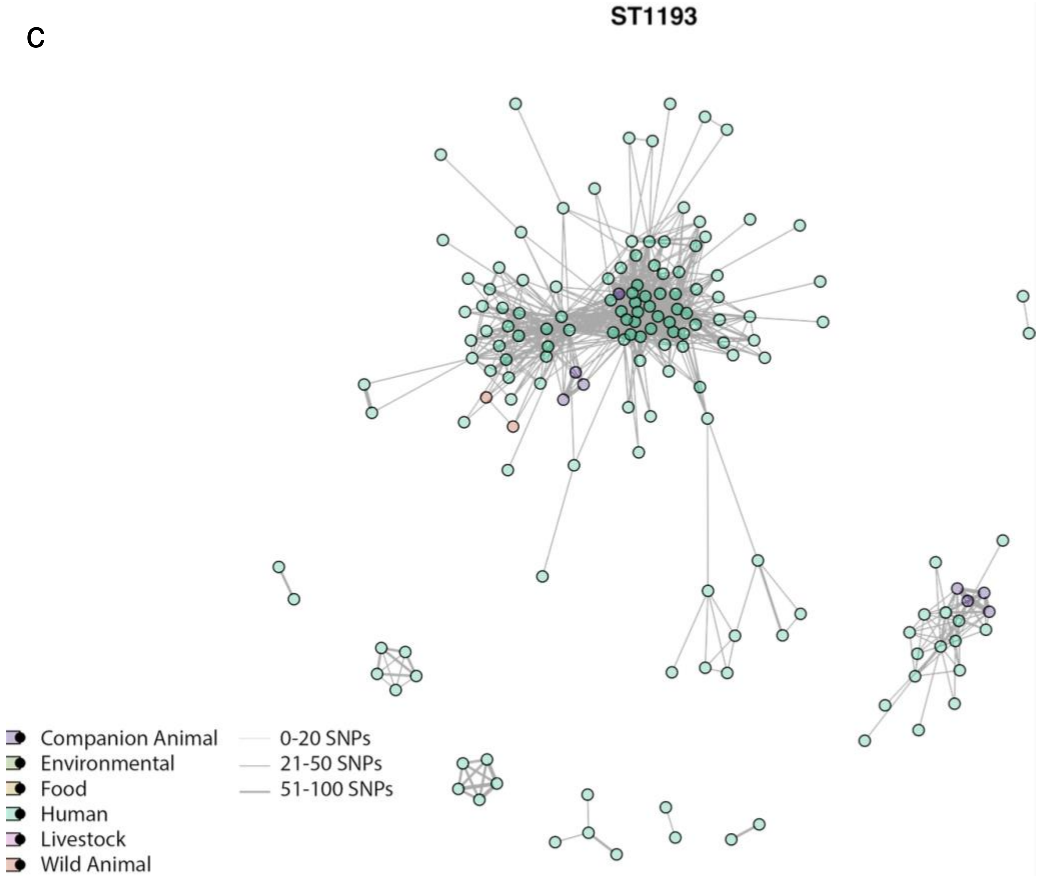
ST1193
Companion Animal
Environmental
Food
Human
Livestock
Wild Animal
0-20 SNPs
21-50 SNPs
51-100 SNPs

d

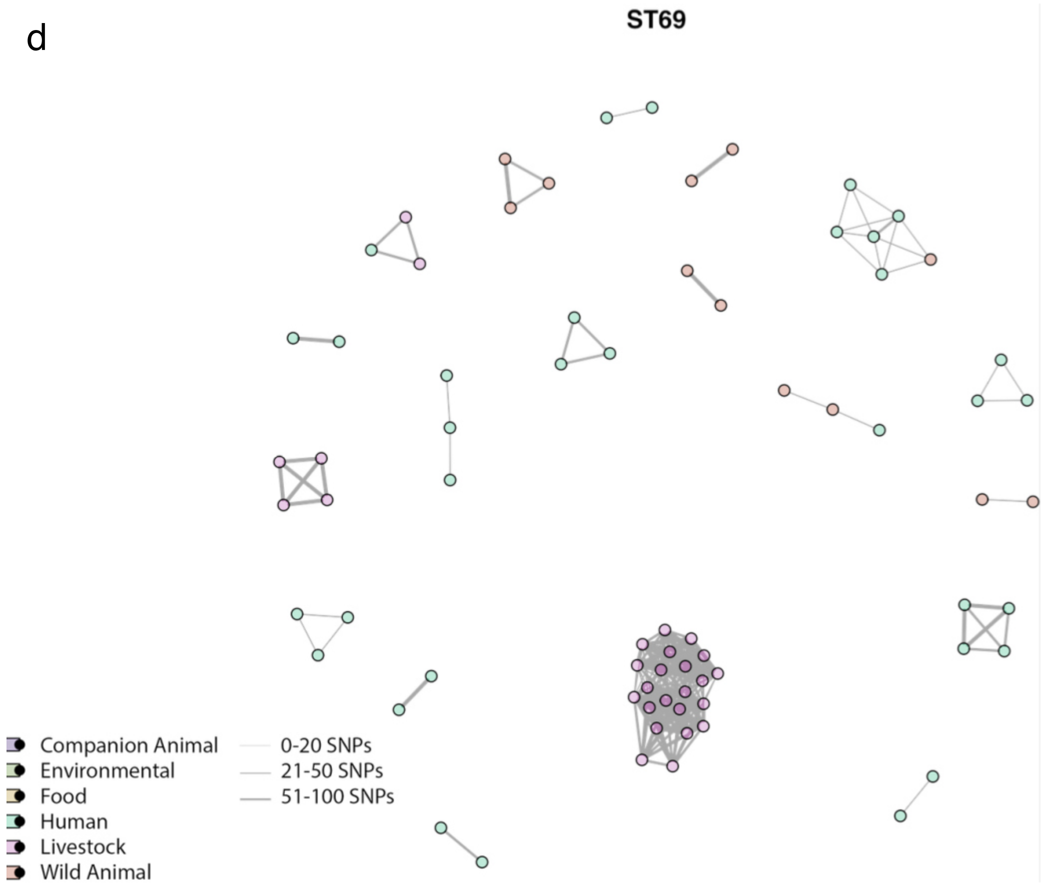
ST69
Companion Animal
Environmental
Food
Human
Livestock
Wild Animal
0-20 SNPs
21-50 SNPs
51-100 SNPs

e

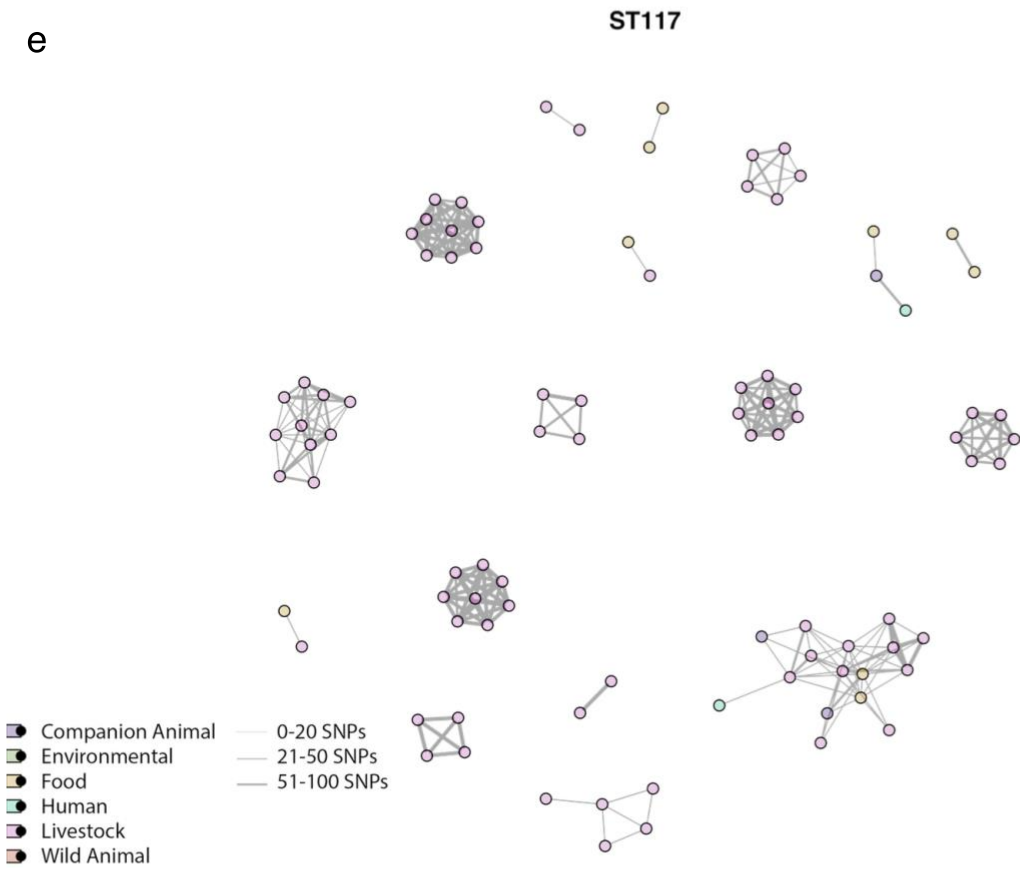
ST117
Companion Animal
Environmental
Food
Human
Livestock
Wild Animal
0-20 SNPs
21-50 SNPs
51-100 SNPs

f

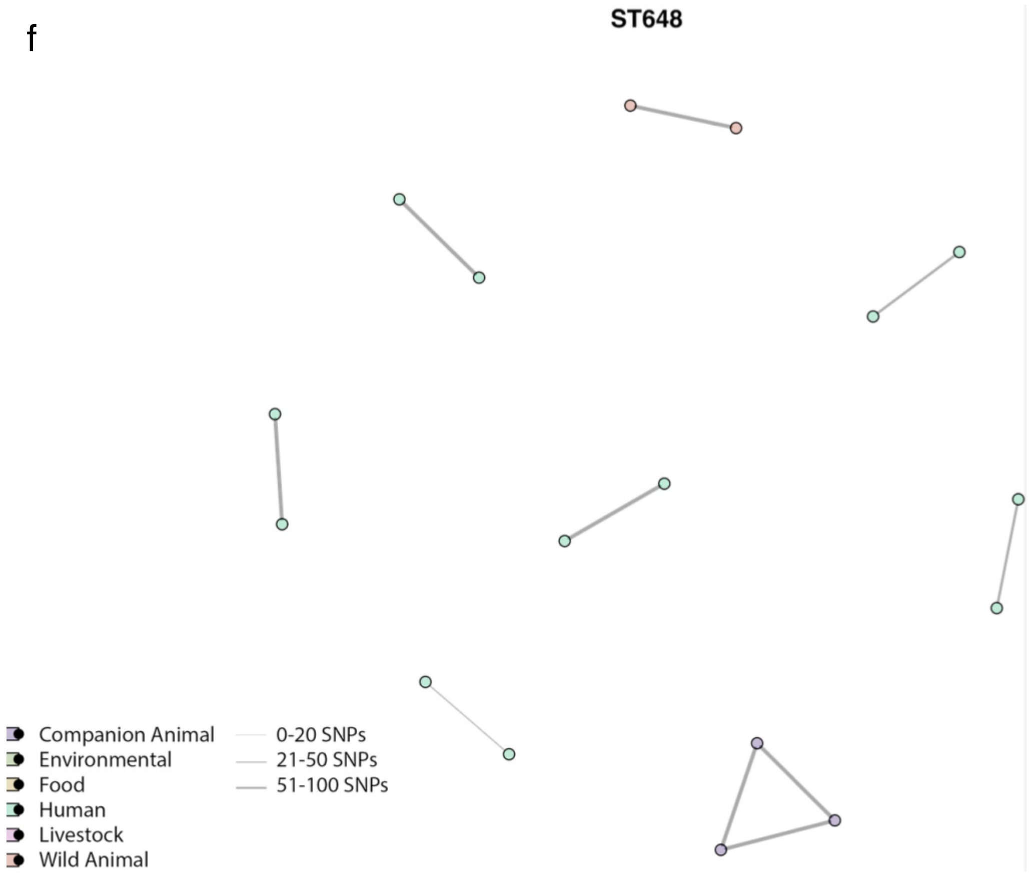
ST648
Companion Animal
Environmental
Food
Human
Livestock
Wild Animal
0-20 SNPs
21-50 SNPs
51-100 SNPs

g

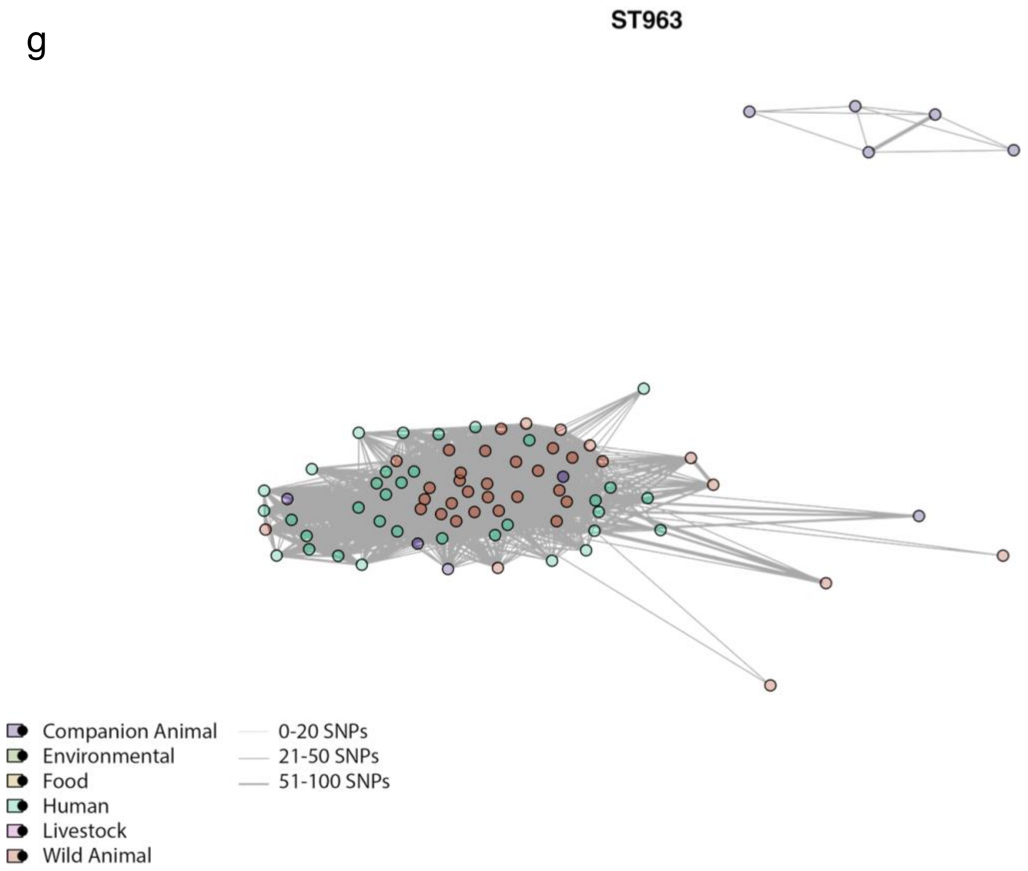

ST963
Companion Animal
Environmental
Food
Human
Livestock
Wild Animal
0-20 SNPs
21-50 SNPs
51-100 SNPs

h

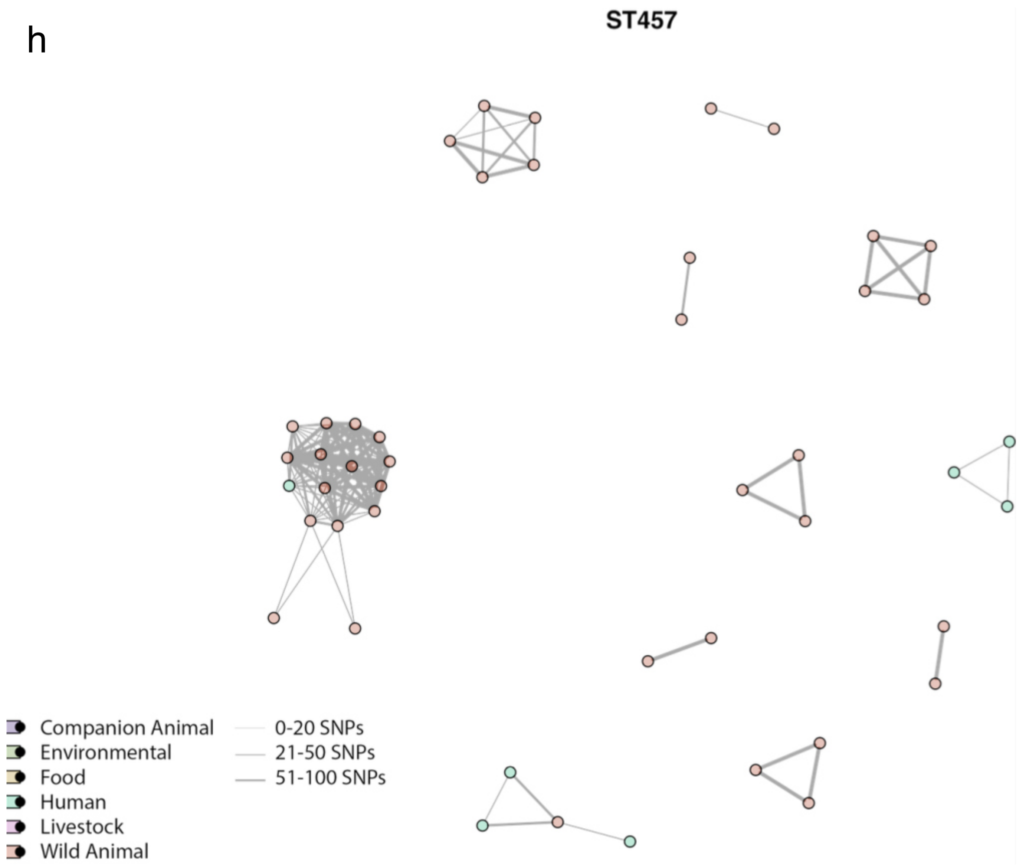

ST457
Companion Animal
Environmental
Food
Human
Livestock
Wild Animal
0-20 SNPs
21-50 SNPs
51-100 SNPs

i

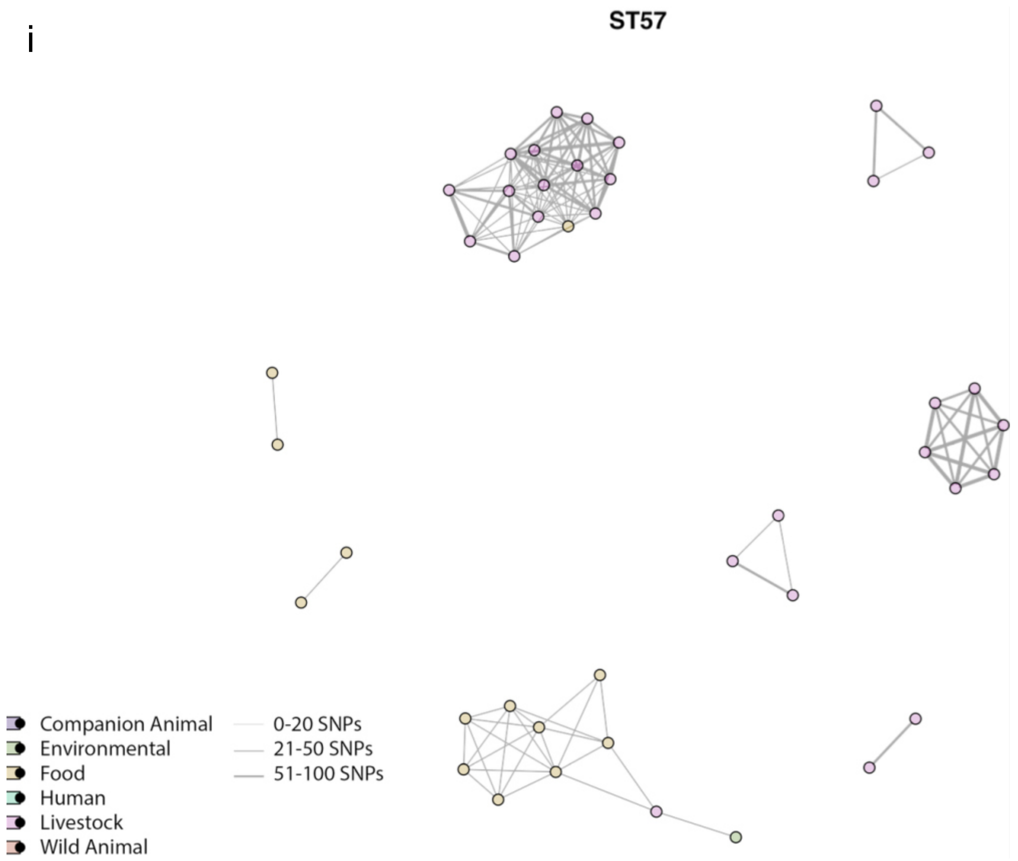
ST57
Companion Animal
Environmental
Food
Human
Livestock
Wild Animal
0-20 SNPs
21-50 SNPs
51-100 SNPs

j

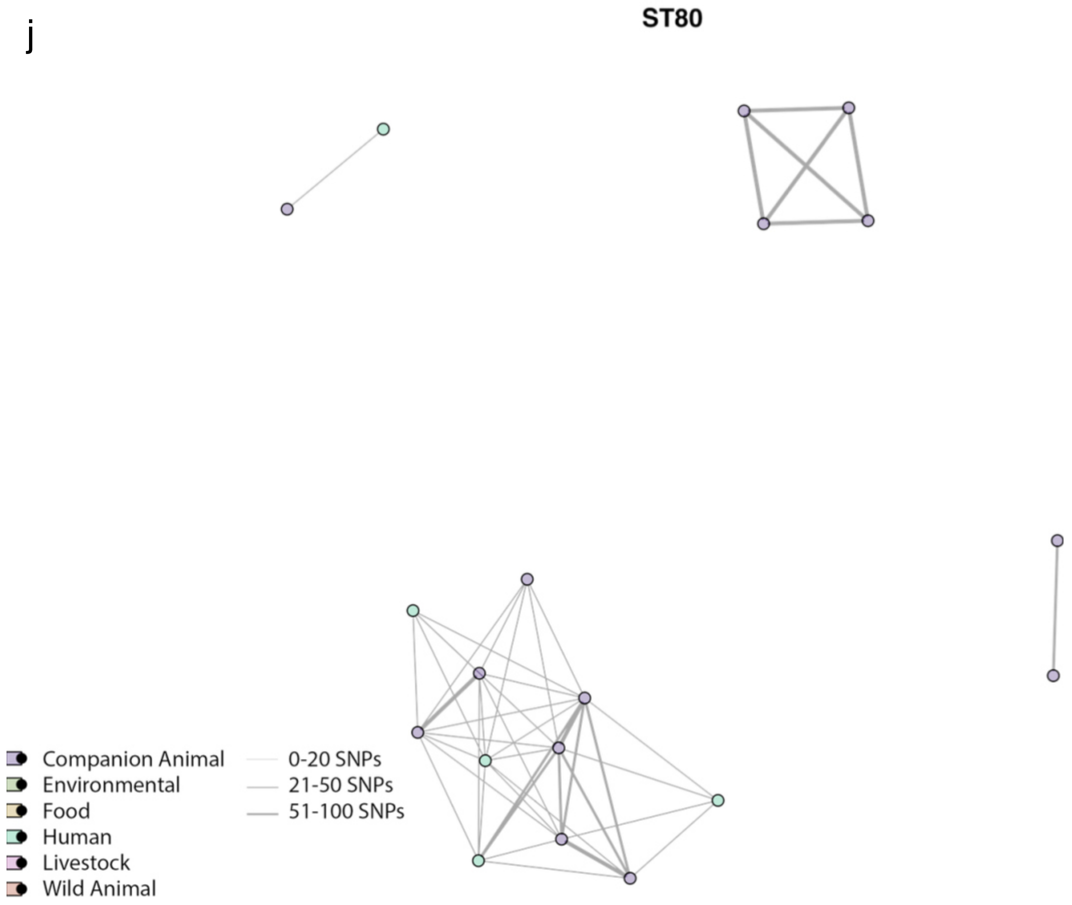
ST80
Companion Animal
Environmental
Food
Human
Livestock
Wild Animal
0-20 SNPs
21-50 SNPs
51-100 SNPs

Supplementary Fig. 2 a-j. Network analyses for 10 STs commonly associated with cross-sectoral concurrences representing their mono-source and cross-source dissemination. Isolates included have a pairwise SNP distance of ≤ 100 SNPs and are coloured by source.

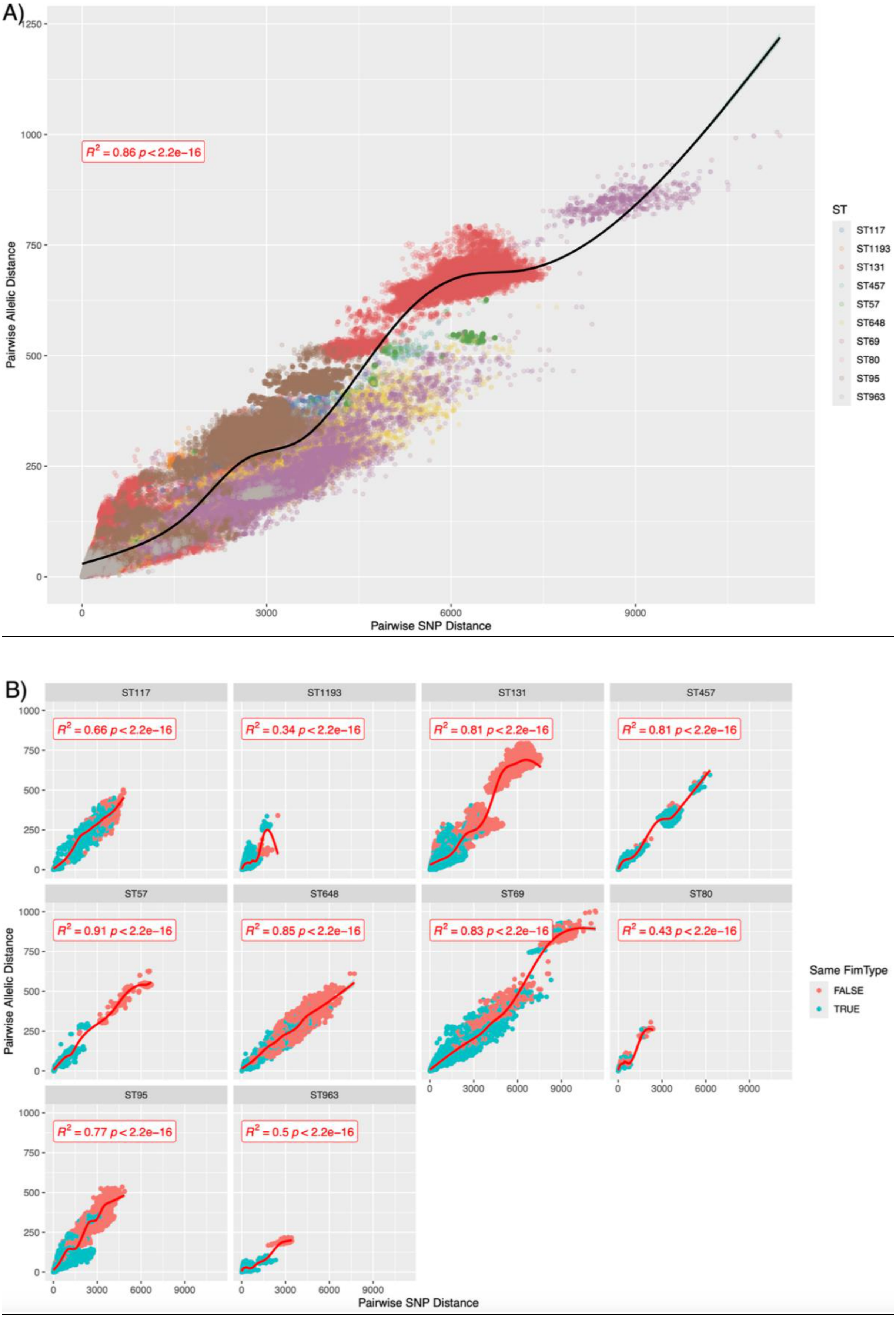
A)
$R^2 = 0.86\ p < 2.2e{-}16$
Pairwise Allelic Distance
Pairwise SNP Distance
ST
ST117
ST1193
ST131
ST457
ST57
ST648
ST69
ST80
ST95
ST963
B)
ST117
$R^2 = 0.66\ p < 2.2e{-}16$
ST1193
$R^2 = 0.34\ p < 2.2e{-}16$
ST131
$R^2 = 0.81\ p < 2.2e{-}16$
ST457
$R^2 = 0.81\ p < 2.2e{-}16$
ST57
$R^2 = 0.91\ p < 2.2e{-}16$
ST648
$R^2 = 0.85\ p < 2.2e{-}16$
ST69
$R^2 = 0.83\ p < 2.2e{-}16$
ST80
$R^2 = 0.43\ p < 2.2e{-}16$
ST95
$R^2 = 0.77\ p < 2.2e{-}16$
ST963
$R^2 = 0.5\ p < 2.2e{-}16$
Pairwise Allelic Distance
Pairwise SNP Distance
Same FimType
FALSE
TRUE

Supplementary Fig. 3. A) Scatter plot visualising the degree of correlation between cgMLSTallelic distance (y-axis) and SNPdistance (x-axis). Data points are coloured by STfor the pair of isolates comprising a given datapoint (only isolates sharing an STare shown). B) Scatter plot visualising the degree of correlation between cgMLSTallelic distance (y-axis) and SNPdistance (x-axis) for 10 sequence types under analysis. Data points are coloured based on whether or not a given pair of isolates shares a fimH type; a proxy for phylogenetic differences which correlates with the clustering profiles for the data presented (only isolates sharing an STare shown).

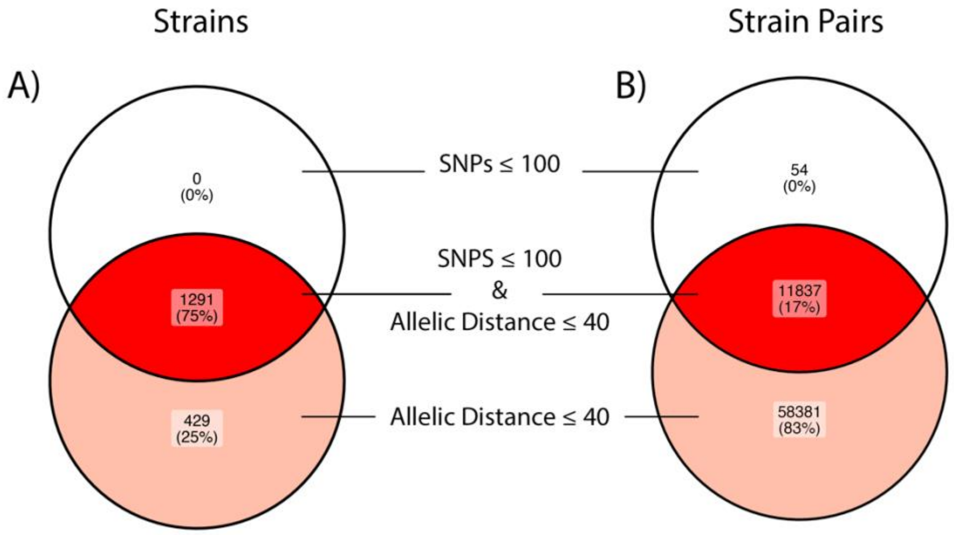

Supplementary Fig. 4. Venn diagram visualising the intersection of putative clusters (as per cgMLST; ≤ 40 allelic differences) and clusters (as per SKA; ≤ 100 SNPs). Only strains of ST117, ST1193, ST131, ST457, ST57, ST648, ST69, ST80, ST95, ST963 are shown. A) Count of strains which exhibited matches to other strains at 100 SNPs or closer (top), 40 or fewer allelic differences (bottom) and those that met both criteria (middle). Note that 228 strains met no such criteria. B) Count of strain pairs that matched at 100 SNPs or closer (top), 40 or fewer allelic differences (bottom) and those that met both criteria (middle).

a

Cluster Quality Metrics for ST131

Network with SNP Distance < 20 | Network with SNP Distance < 100

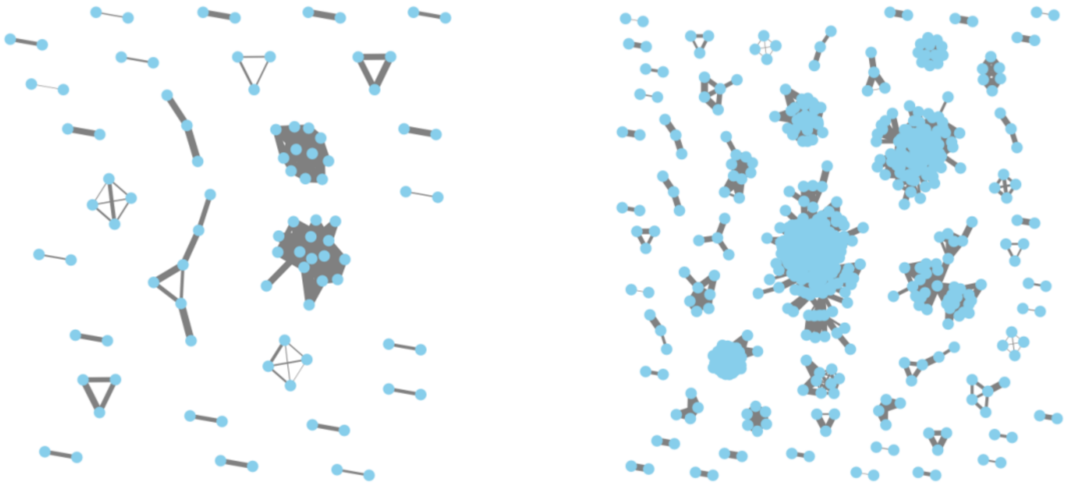

Metric	Observed Difference	p-value
Silhouette	0.1854	0.0000
Calinski-Harabasz	-3727.0280	0.0000
Davies-Bouldin	-1.0147	1.0000
WCSS	-2001003042.0467	0.0000
Cohesion	-1419331.9857	0.0000
Separation	-1418.6668	1.0000

b

Cluster Quality Metrics for ST95

Network with SNP Distance < 20 | Network with SNP Distance < 100

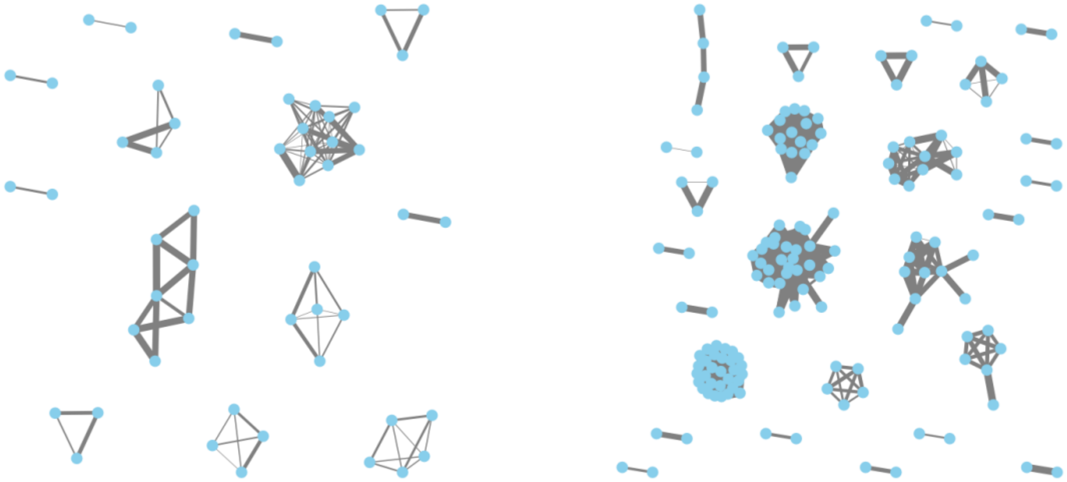

Metric	Observed Difference	p-value
Silhouette	0.1590	0.0000
Calinski-Harabasz	-782.2758	0.0000
Davies-Bouldin	-0.3770	0.0010
WCSS	-58685857.6525	0.0000
Cohesion	-310981.3034	0.2880
Separation	-539.9365	1.0000

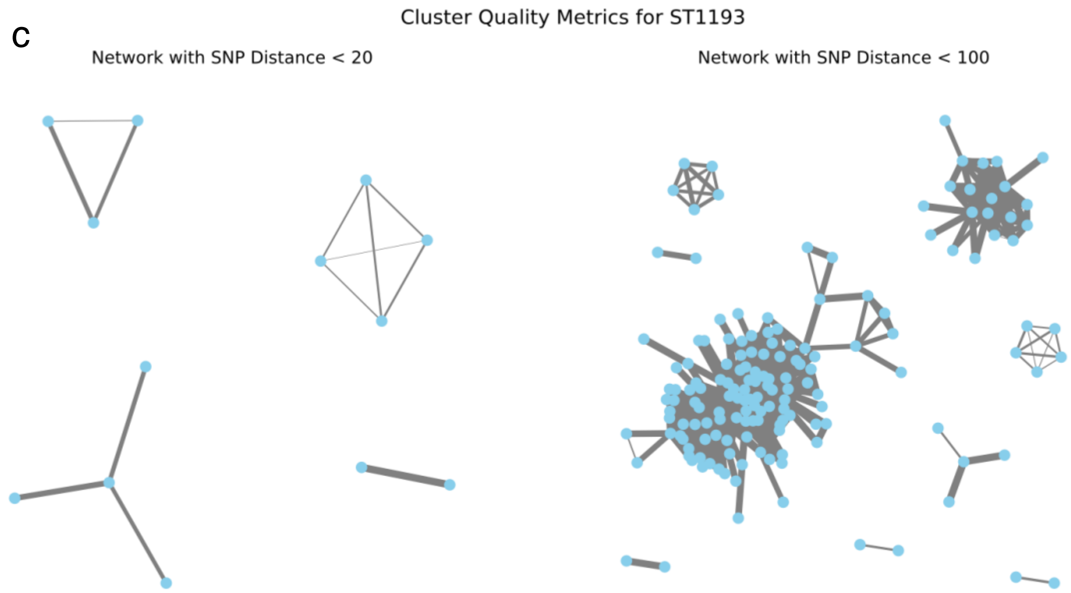

Metric	Observed Difference	p-value
Silhouette	0.1624	0.0000
Calinski-Harabasz	-301.9431	0.0000
Davies-Bouldin	-1.2680	1.0000
WCSS	-65958871.9667	0.0000
Cohesion	-40185.8802	0.0000
Separation	-32.8812	1.0000

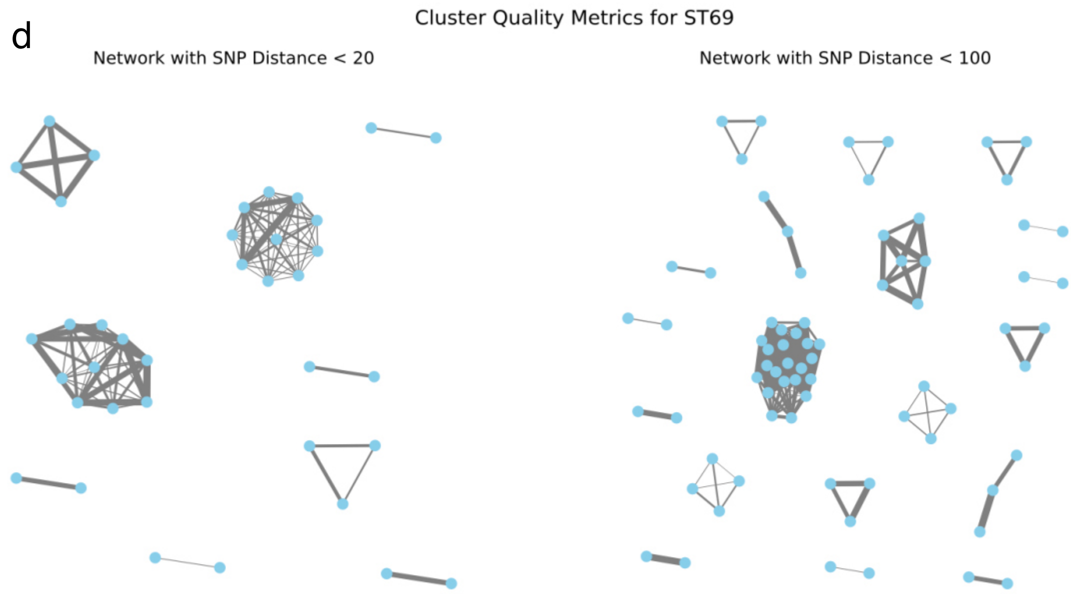

Metric	Observed Difference	p-value
Silhouette	0.1702	0.4810
Calinski-Harabasz	-1314.1863	0.0000
Davies-Bouldin	-0.1221	0.0040
WCSS	-22167329.8083	0.0000
Cohesion	-125751.0176	0.4710
Separation	430.3998	1.0000

e

Cluster Quality Metrics for ST117

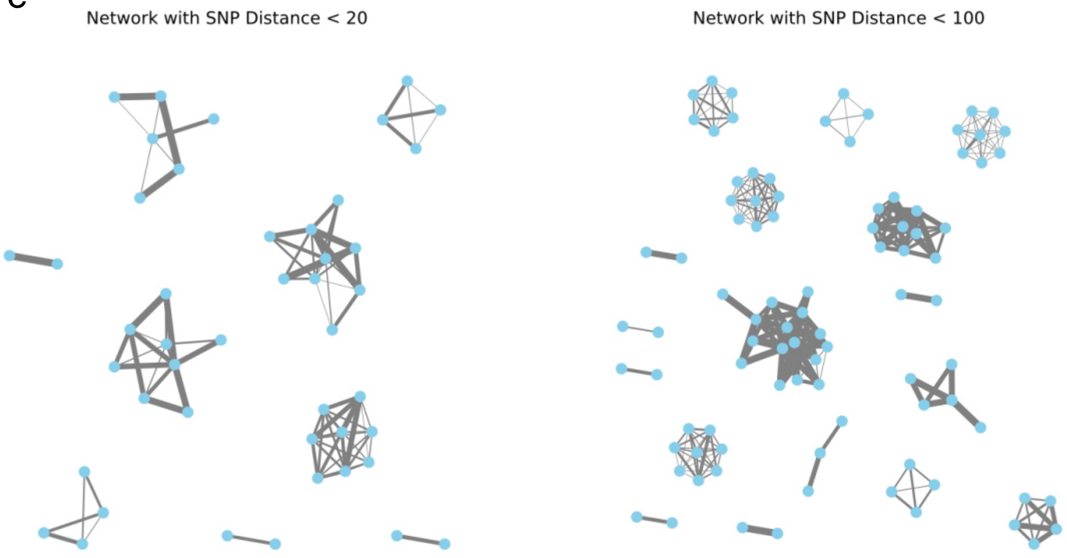

Metric	Observed Difference	p-value
Silhouette	0.3303	0.9960
Calinski-Harabasz	-5389.8228	0.0000
Davies-Bouldin	-0.1555	0.1130
WCSS	-24834836.9833	0.0000
Cohesion	-227303.1209	1.0000
Separation	-275.6874	1.0000

f

Cluster Quality Metrics for ST648

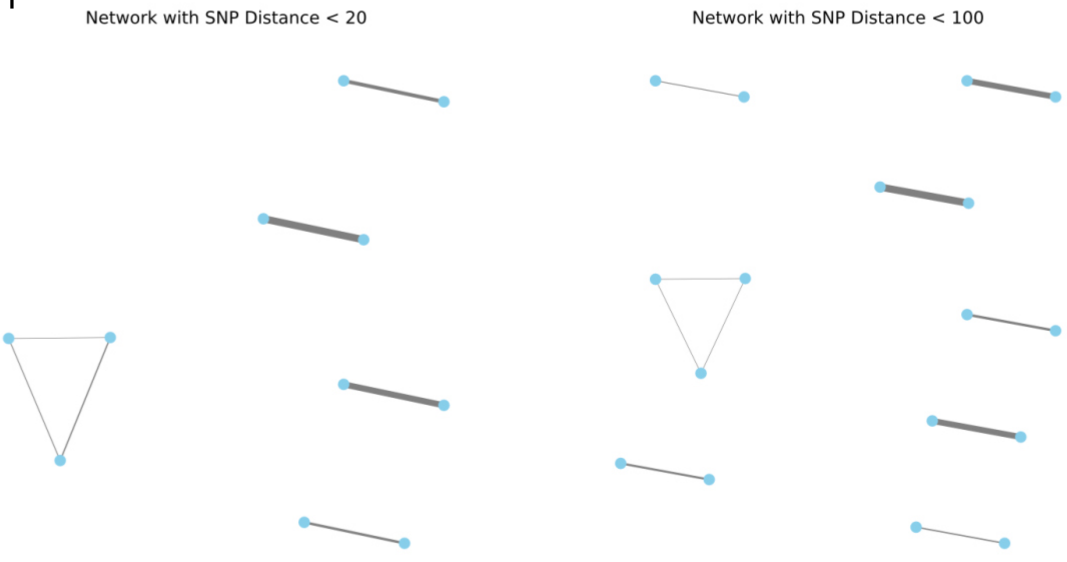

Metric	Observed Difference	p-value
Silhouette	0.0326	0.6130
Calinski-Harabasz	1768.9853	1.0000
Davies-Bouldin	-0.0058	0.0870
WCSS	-21995.5000	0.0870
Cohesion	-10299.8276	0.7090
Separation	-14.2851	0.9130

g

Cluster Quality Metrics for ST57

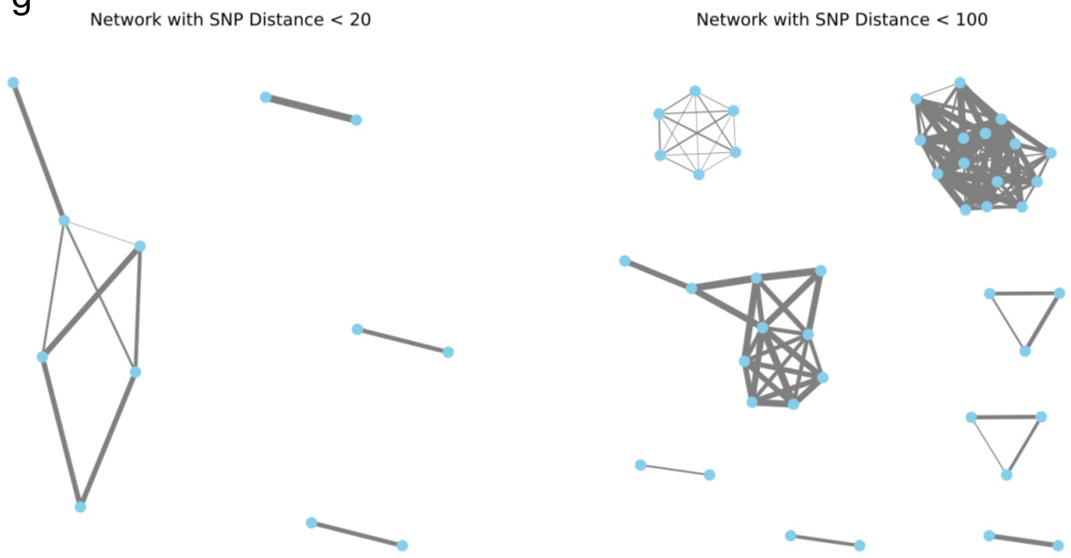

Metric	Observed Difference	p-value
Silhouette	0.3766	0.3500
Calinski-Harabasz	-180779.8566	0.0000
Davies-Bouldin	-0.2089	0.0000
WCSS	-2159303.7000	0.0000
Cohesion	-212447.7261	1.0000
Separation	-4730.7306	0.2380

h

Cluster Quality Metrics for ST80

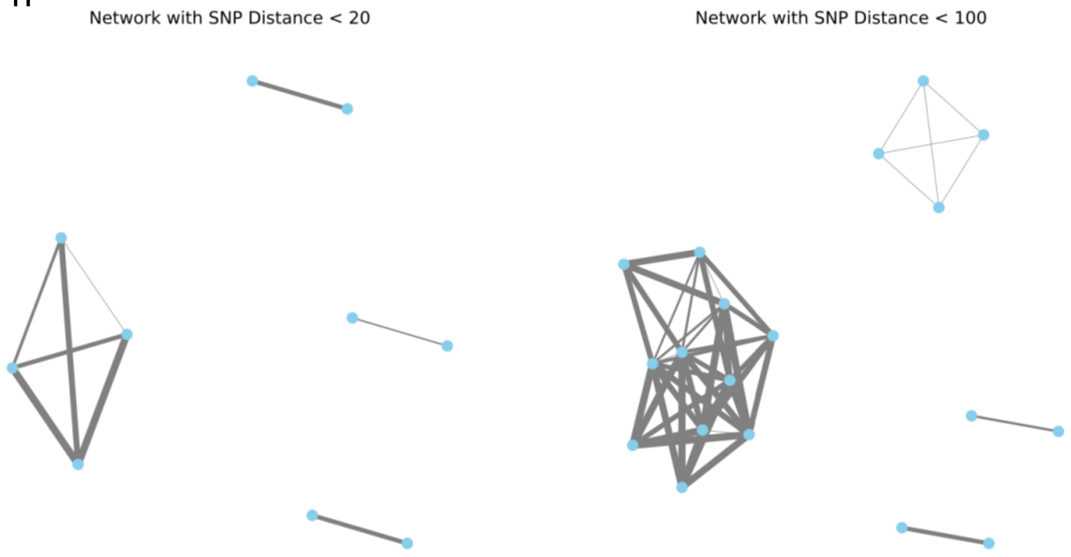

Metric	Observed Difference	p-value
Silhouette	0.0688	0.0520
Calinski-Harabasz	-389.4906	0.0000
Davies-Bouldin	-0.4237	0.3680
WCSS	-2799825.3333	0.0000
Cohesion	-5206.3043	0.2180
Separation	256.9336	1.0000

i

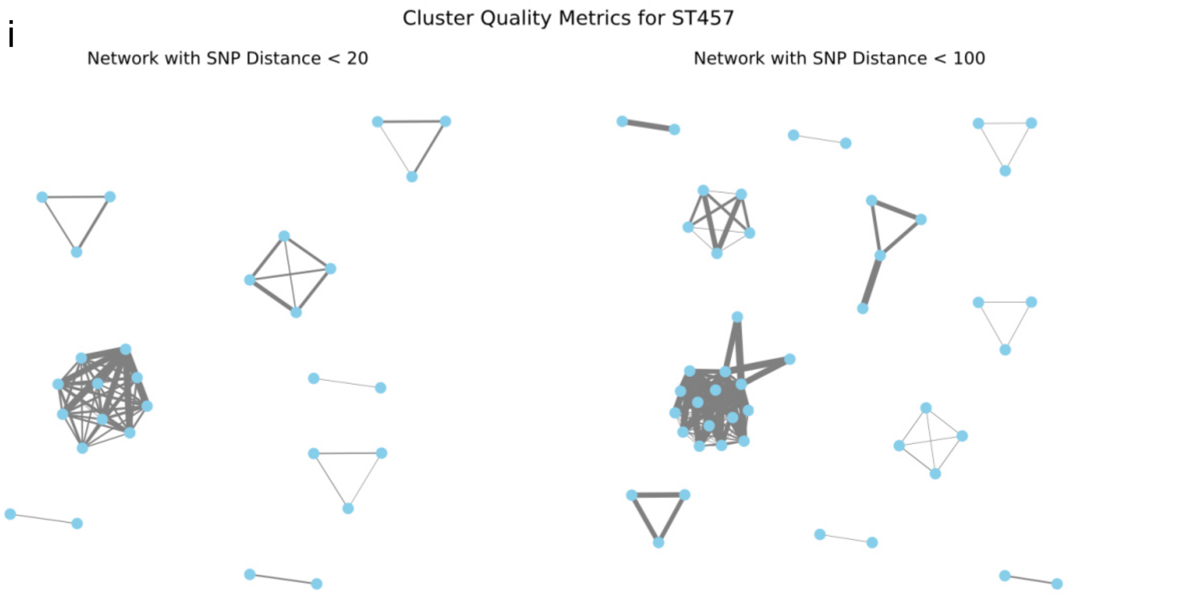

Metric	Observed Difference	p-value
Silhouette	0.1331	0.6340
Calinski-Harabasz	-74595.8971	0.0000
Davies-Bouldin	-0.2578	0.7230
WCSS	-5577706.3833	0.0010
Cohesion	-55079.8475	0.9930
Separation	7.4104	1.0000

Supplementary Fig. 5 a-i. Cluster metric analysis of different cluster cut-off thresholds for each ST. The difference in each metric (Silhouette, Calinski-Harabasz, Davies-Bouldin, Within-Cluster Sum of Squares, Cohesion, and Separation) was calculated for both thresholds (≤ 20 and ≤ 100) and is reported as the observed difference. A positive observed difference favours the 100 SNP threshold. The p-value for the observed difference was determined via permutation test (1,000 iterations).

Supplementary Methods and Notes

Additional Preliminary Clustering Analysis

We performed an additional preliminary clustering step using PopPunk to assess the intersection of PopPunkderived genomic clusters and those identified using ≤40 cgMLST allelic distance and ≤100 SNPdistance thresholds.

Among all genomes under analysis total of 608 PopPunkclusters were identified, whilst cgMLSTidentified 591 distinct clusters. All genomes which formed a PopPunk cluster were found to differ by ≤ 40 cgMLSTalleles. Strains which differed in PopPunk clusters had as few as 203 allelic differences; those which shared a PopPunkcluster exhibited as many as 1,184 allelic differences.

Among the 10 STs analysed in detail (131, 963, 1193, 95, 69, 80, 117, 457, 648 and 57), 129 clusters were identified using cgMLST,while 19 were identified using PopPunk. All isolates exhibiting 100 SNPsor fewer were of the same PopPunk cluster.

Our SNP clusters (at a threshold of ≤ 100 SNPs)therefore includes much more closely related genomes than do PopPunk clusters as does our approach using cgMLST. This is consistent with findings of Lees et al, wherein genomes sharing a PopPunkcluster differ by up to 1,000 cgMLSTallelic differences.

Supplementary Analysis 2 - Clustering of Regionally and Temporally Restricted Genomes

We analysed a subset our collection from the same region during a shorter time-period to assessfor potential transmission events within this time-period. While high resolution metadata pertaining to sample collection suitable for epidemiological analysis is unavailable for the present collection, genomes located more closely in space and time are stronger candidates for potential transmission events which we sought to explore. We therefore selected genomes collected in Victoria between 2013-2017, of which 906 genomes were identified. This period and region were chosen due to it having a higher representation of genomes than other potential sampling windows (Supplementary Fig.

6). Following detailed metadata curation, we removed an additional 120 poultry-associated genomes, leaving a remainder of 786, due to their having been isolated from different anatomical sites within the same poultry hosts suffering from invasive extraintestinal infections and thus being potential clones.

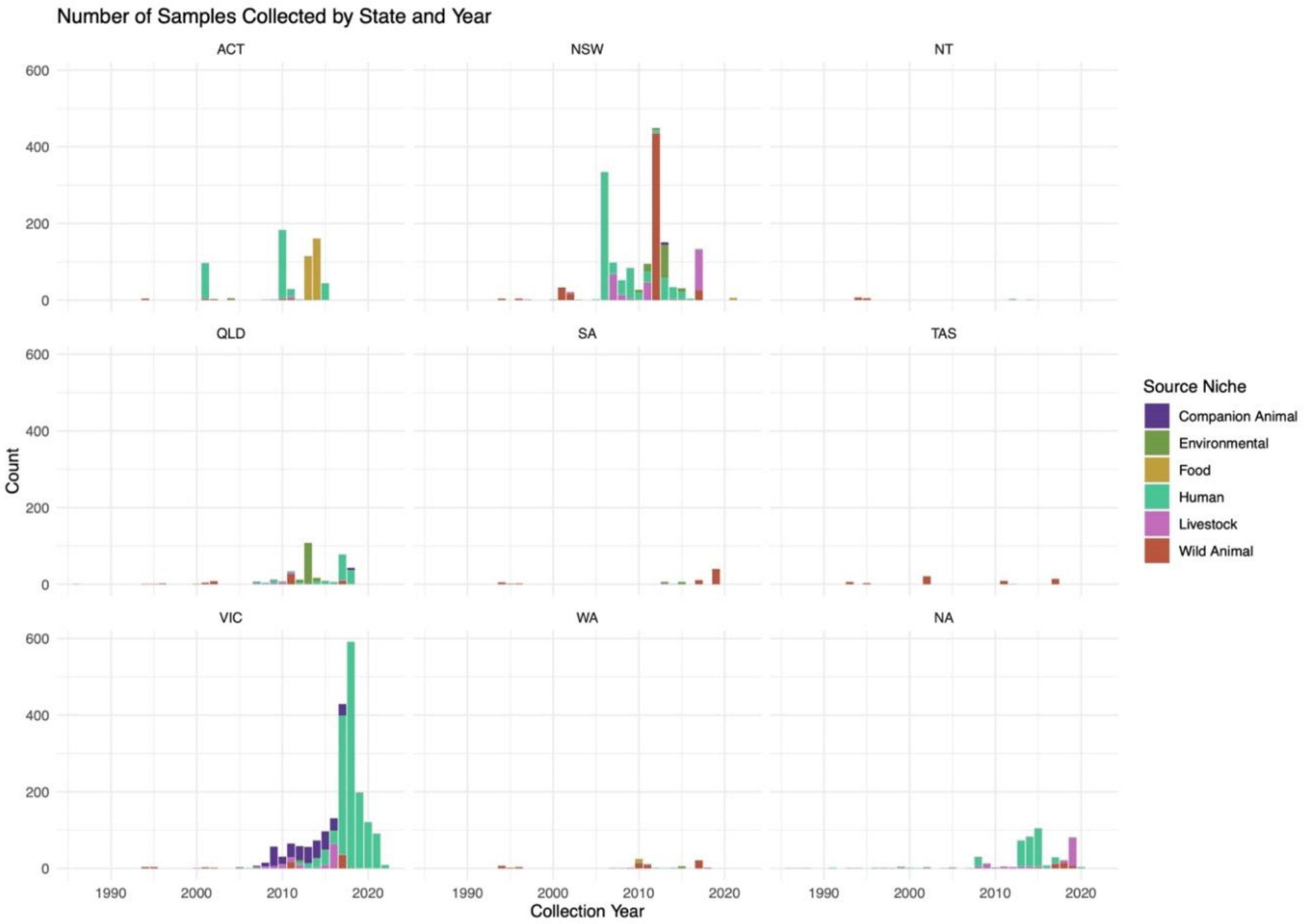

Supplementary Fig. 6 - Collection Composition by Region(State), Year and Source. This figure visualises the collection by State with year of collection shown on the x-axis, count of genomes shown on the y-axis, and stacked bars coloured by source of isolation. ACT–Australian Capital Territory, NSW–New South Wales, NT–Northern Territory, QLD–Queensland, SA–South Australia, TAS–Tasmania, VIC–Victoria, WA–Western Australia, NA–State of origin unknown

Within this sampling window, we identified cross-source clusters across 4/10 STs (ST963, ST131, ST95 and ST69) with pairs differing by as few as 40 SNPs, and single-source clusters among 9/10 STs (ST963, ST131, ST95, ST69, ST80, ST648, ST57, ST117 and ST1193) with pairs differing by as few as 0 SNPs (Supplementary Fig. 7). Note however that cross-source clusters could not be observed for STs 57, 457 and 80 as within such STs in this sampling period only a single source was represented.

Future studies focusing on a snapshot dataset of isolates collected from the same region in a shorter time period than our present study (which includes isolates spanning a 36-year period) will likely find a greater proportion of potential transmission events and provide greater insight into the frequency of these events within a One Health context.

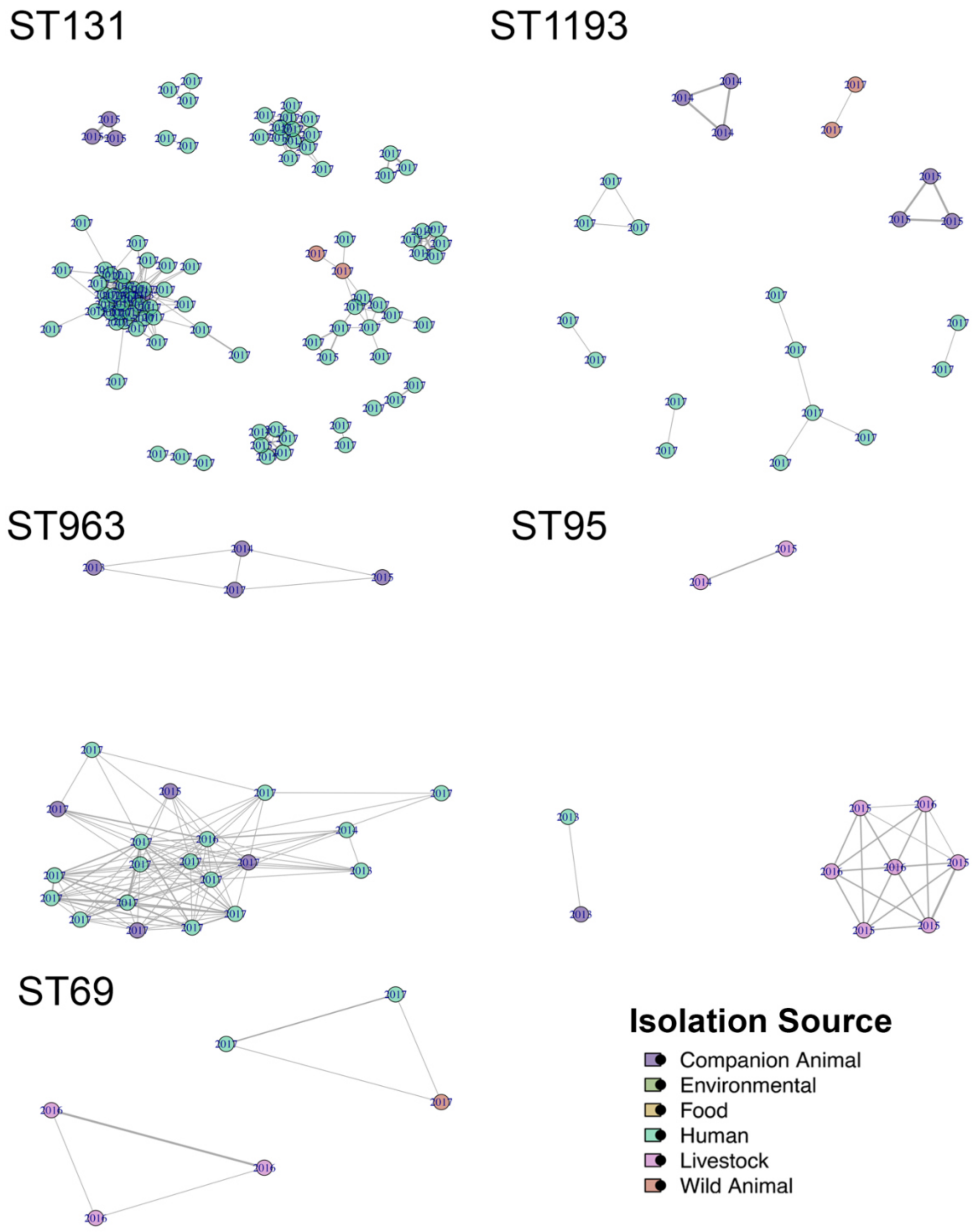
ST131
ST1193
ST963
ST95
ST69
Isolation Source
Companion Animal
Environmental
Food
Human
Livestock
Wild Animal

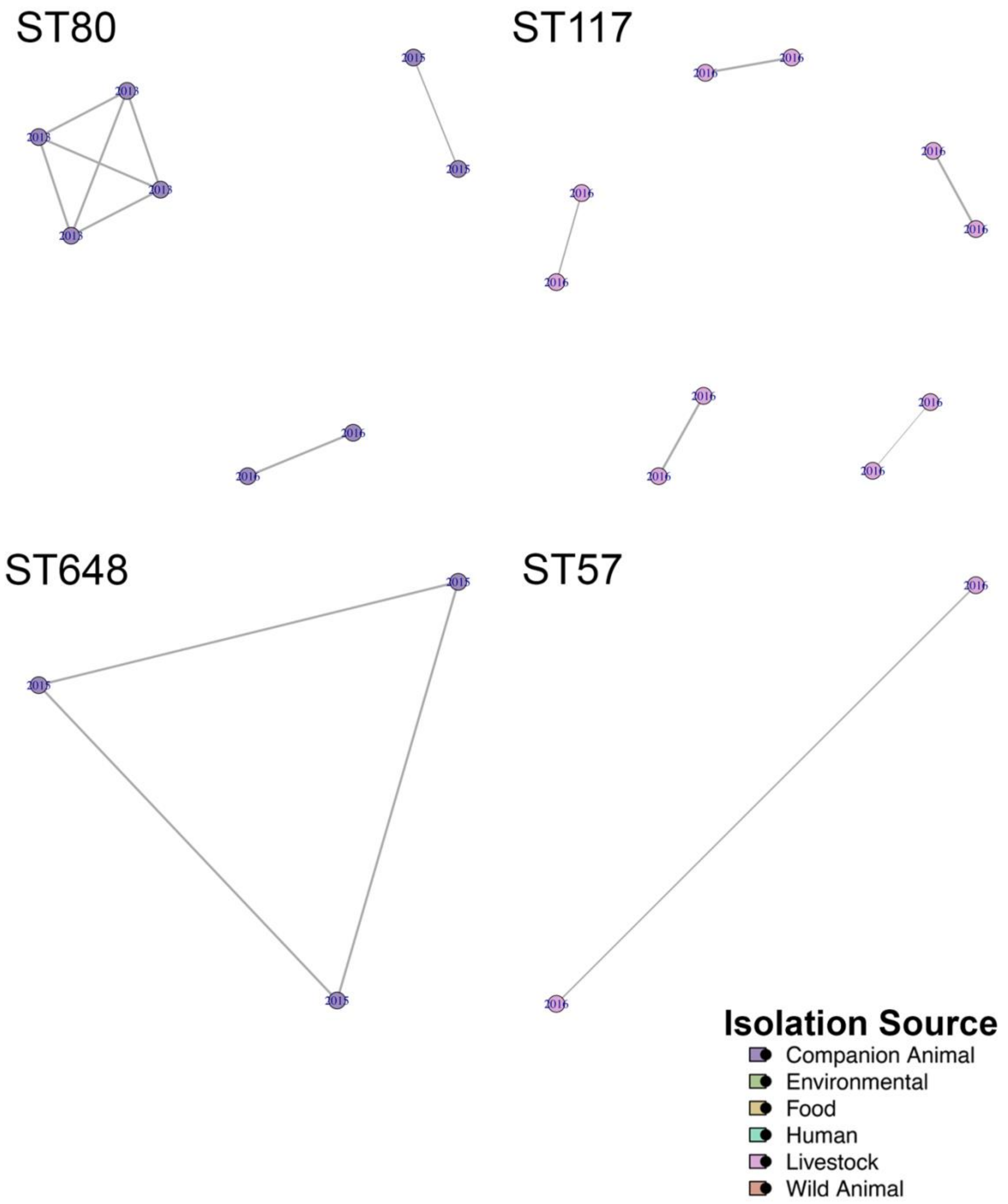

Supplementary Fig. 7 - Network analyses for STs commonly associated with multi-sectoral clusters representing their intra-source and inter-source dissemination. Clusters include two or more instances of genomes which have a pairwise SNP distance of ≤ 100 SNPs and are nodes representing genomes are coloured by their isolation source and labelled with their year of isolation. This analysis contains only a subset of genomes from the same Australian State (Victoria, Australia) and collected within a five-year period. Analyses were performed for STs 131, 963, 1193, 95, 69, 80, 117, 457, 648 and 57

(note that STs 457 and 648 are not shown due to a lack of clustering of strains from this sampling window). SNP distances for clusters is available in Supplementary Data 2.

Supplementary Analysis 3 - Clustering of Potential Transmission Events among an External Genome Collection

We analysed a collection of 1,338 E. coli from Kenya by Muloi et al (2022) which includes detailed metadata on the individual households from which E. coli sourced from humans and their household livestock. Samples were collected between 2015 and 2016 from 99 households in Nairobi, Kenya (an area of approximately 700km^2). The motivation for this supplementary analysis is to assess the capacity for our approach to detect potential transmission events in a dataset containing high resolution metadata and genomes from isolates which are reasonably likely to constitute cross-source transmission events.

In this analysis we identified 207 pairs of strains which differ by 100 or fewer SNPs, with some pairs differing by as few as 2 SNPs (Supplementary Data 4). In total, spanning 83 STs were identified which met such criteria. Of these, 67/207 pairs were sourced from different host types. A total of 25/67 were collected from the same households, and among the 42/207 collected from different households, 10 were collected from different households in the same region while 32 were collected from households in different regions.

Among strains originating from different host types, we identified examples differing by as few as 4 SNPs. The combination of epidemiological data and the close phylogenetic relatedness of these samples suggests these constitute transmission events. Our data set, being opportunistically aggregated, does not contain such depth of metadata, however this analysis suggests that our approach, when combined with high quality epidemiological metadata, may even be useful in identifying transmission events.

set, being opportunistically aggregated, does not contain such depth of metadata, however this analysis suggests that our approach, when combined with high quality epidemiological metadata, may even be useful in identifying transmission events.

Supplementary Data Captions

Supplementary Data 1

Details the metadata, accession numbers, bioproject IDs, sequence types, plasmid sequence types and cluster IDs for genomes under analysis.

Supplementary Data 2

Details metadata and phylogenetic distances (including both SNP and cgMLST allelic distances) between pairs of genomes under analysis from the ten sequence types which were analysed in greater detail.

Supplementary Data 3

Summarises the counts of strain pairs visualised in box plots among combinations of sequence types and sources in Figure 5.

Supplementary Data 4

Details summary statistics for SNP distances among clusters of sequence types analysed from Muloi et al (2022).
