# Supplementary material for: Parameters for one health genomic surveillance of Escherichia coli from Australia
Source: Nat Commun. 2025 Jan 2;16:17. doi: 10.1038/s41467-024-55103-2 (PMC11696363; doi:10.1038/s41467-024-55103-2)
Supplement: Supplementary file 2 — Description of Additional Supplementary Files [file 41467_2024_55103_MOESM2_ESM.pdf]

Description of Additional Supplementary Files

File Name: Supplementary Data 1
Description: Details the metadata, accession numbers, bioproject IDs, sequence types, plasmid sequence types and cluster IDs for genomes under analysis.

File Name: Supplementary Data 2
Description: Details metadata and phylogenetic distances (including both SNP and cgMLST allelic distances) between pairs of genomes under analysis from the ten sequence types which were analysed in greater detail.

File Name: Supplementary Data 3
Description: Summarises the counts of strain pairs visualised in box plots among combinations of sequence types and sources in Figure 5.

File Name: Supplementary Data 4
Description: Details summary statistics for SNP distances among clusters of sequence types analysed from Muloi et al (2022).
